# Therapeutic hypothermia after cardiac arrest increases the plasma level of B-type natriuretic peptide

**DOI:** 10.1038/s41598-020-72703-2

**Published:** 2020-09-23

**Authors:** Yusuke Kashiwagi, Kimiaki Komukai, Haruka Kimura, Toraaki Okuyama, Tomoki Maehara, Keisuke Fukushima, Takahito Kamba, Yoshitsugu Oki, Keisuke Shirasaki, Takeyuki Kubota, Satoru Miyanaga, Tomohisa Nagoshi, Michihiro Yoshimura

**Affiliations:** 1 Division of Cardiology, Department of Internal Medicine, Kashiwa Hospital, The Jikei University School of Medicine, Kashiwa, Chiba Japan; 2 Division of Cardiology, Department of Internal Medicine, The Jikei University School of Medicine, 3-25-8, Nishi-shimbashi, Minato-ku, Tokyo, 105-8461 Japan

**Keywords:** Myocardial infarction, Heart failure, Diagnostic markers

## Abstract

Natriuretic peptides (NPs) regulate blood pressure and fluid homeostasis and exert various effects on the cardiovascular system. Recently, the relationship between NPs and the energy metabolism has been reported, and using a cell culture experiment system, we previously showed that NP activated brown cells in a low temperature environment while also suppressing a decrease in the cell temperature. However, few reports have described the secretion of NPs in cold environments, and there have been almost no studies of B-type natriuretic peptide (BNP) in humans. We investigated how NPs respond to cold environments in 21 patients who underwent therapeutic hypothermia (TH) after cardiac arrest. The plasma BNP levels were significantly increased (more than fivefold) during TH (logarithmically from 1.98 ± 0.79 to 2.63 ± 0.59, P < 0.01). During TH, diastolic pulmonary artery pressure (PAP) significantly decreased, and there were no significant changes in the stroke volume index (SVI). This increase of BNP was not associated with any hemodynamic changes. In contrast to our findings for BNP, the change in A-type NP (ANP) was quite small. We detected a significant increase in the plasma BNP levels during TH, unrelated to hemodynamics. This elevation of BNP levels seems to be potential influenced by hypothermia.

## Introduction

Natriuretic peptides (NPs), such as A-type or atrial (ANP) and B-type or brain NP (BNP), are produced and secreted in the heart, and their plasma levels reflect the degree of heart failure^1–6^. BNP is mainly secreted from the ventricle, and its plasma concentration is higher than that of ANP under conditions of heart failure^3,7^. Although it is well known clinically that myocardial stretch is involved in the production and secretion of BNP^8^, its molecular mechanism is still being studied.

Many studies have explored the actions of NPs. Due to the inhibition of renin–angiotensin–aldosterone and the sympathetic nervous systems, NPs regulate the blood pressure and fluid volume through various biological effects such as vasodilation and diuresis, and improve cardiac remodeling^9,10^. At the molecular level, NPs exert their effects via the common receptor guanylyl cyclase (GC)-A, which catalyzes the synthesis of cGMP, leading to the activation of protein kinase (PK) G^11^. Recent evidence indicates that NP/cGMP-dependent protein kinase cascades also regulate the energy balance and glucose homeostasis^12^. We also demonstrated the thermogenic actions of NP in rat brown adipocytes through the p38 mitogen-activated protein kinase (MAPK)-uncoupling protein 1 (UCP1) pathway by increasing the intracellular temperature^13^. These findings suggested that NPs have a novel self-protective function when the core body temperature or the local tissue (including the heart itself) temperature decreases to a level that results in severe hemodynamic conditions, such as serious heart failure.

Patients with severe heart failure experience a decrease in their body temperature due to clinical hypoperfusion^14,15^. Thermal therapy increases the cardiac output and thereby improves the peripheral perfusion in patients with heart failure^16^. However, in humans, the relationship between endogenous NPs and a cold environment remains unclear. NPs may increase under cold conditions, but few clinical data are available concerning ANP^17–19^, and none are available concerning BNP.

Therapeutic hypothermia (TH), which is performed in unconscious survivors with cardiac arrest, reduces the core body temperature to induce neuroprotective effects^20,21^. In this study, we investigated how BNP is affected by a cold environment in patients who underwent TH.

## Results

During the trial period, 24 patients were treated with TH; 2 were excluded due to the administration of steroids before or during TH, and 1 was excluded due to the administration of carperitide during TH. Therefore, 21 patients [male, n = 18 (86%); female, n = 3 (14%); average age 61.2 ± 14.2 years] were ultimately included in the primary analysis.

**Figure 1:**
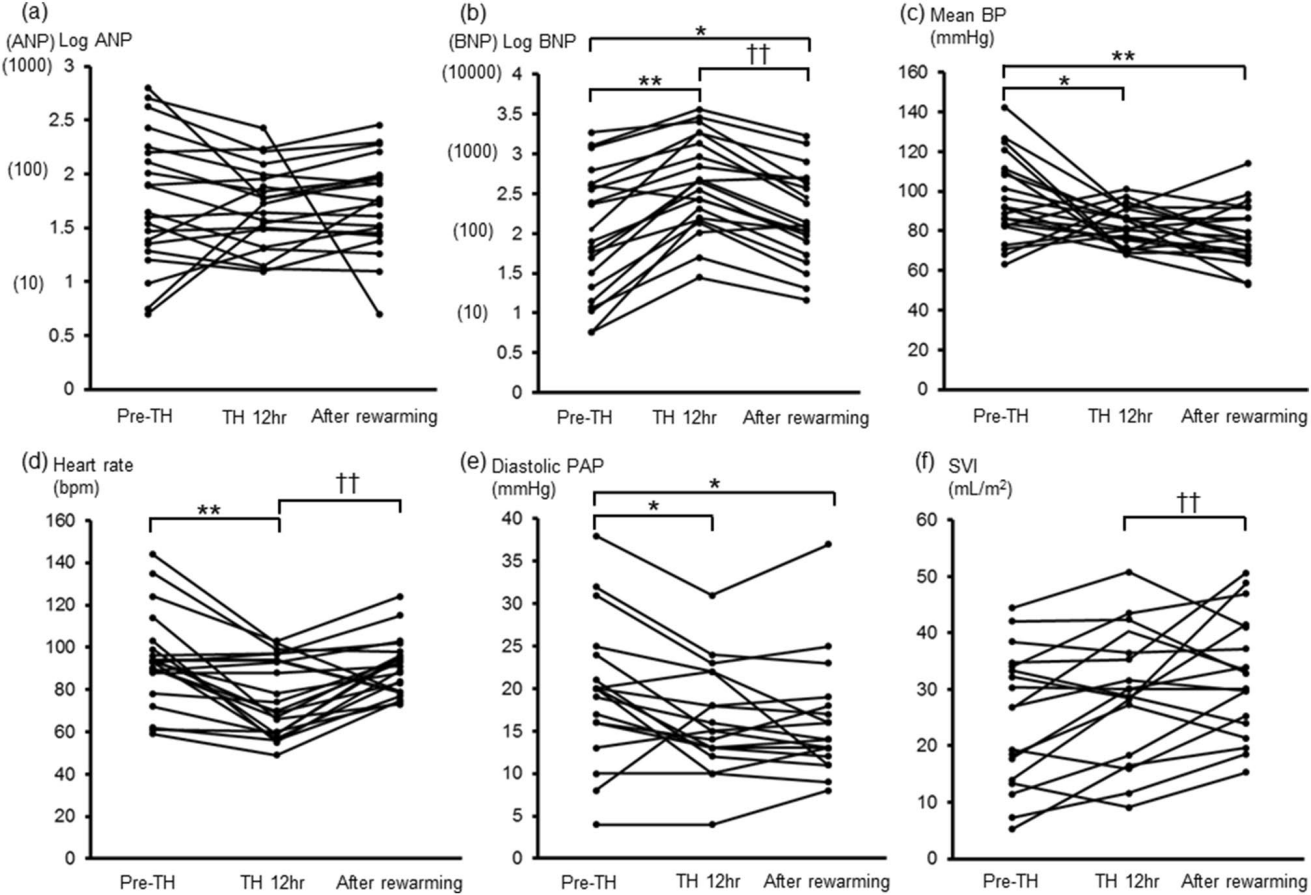
Time course of natriuretic peptide levels and hemodynamic parameters during TH. **(a)** Logarithm (log)-ANP, **(b)** log-BNP, **(c)** mean BP, **(d)** heart rate, **(e)** diastolic PAP, **(f)** SVI. *TH* therapeutic hypothermia, *TH 12 h* 12 h after initiation of therapeutic hypothermia. *ANP* A-type or atrial natriuretic peptide, *BNP* B-type or brain natriuretic peptide, *BP* blood pressure, *PAP* pulmonary artery pressure, *SVI* stroke volume index, * P < 0.05, ** P < 0.01 vs. Pre-TH, † P < 0.05 vs. TH 12 h.

The patients’ background data included their age, gender, cause of cardiac arrest, detailed comorbidities, and outcome in this hospitalization (Table 1). At the time of admission, the hemoglobin (Hb) level, estimated glomerular filtration rate (eGFR), and left ventricular ejection fraction (LVEF) measured by a transthoracic echocardiogram (TTE) were 13.7 ± 2.6 g/dL, 52.3 ± 23.6 mL/min/1.73 m^2^, and 35.7% ± 14.7%, respectively. The median duration in the coronary care unit (CCU) was 10 (7, 14) days. Table 2 shows the medicine and mechanical support administered to each patient during TH. Table 3 shows the clinical data during TH. The plasma BNP levels and logarithm (log)-BNP levels were significantly increased (more than fivefold) during TH [from 80 (21, 400) pg/mL to 450 (156, 1359) pg/mL, P < 0.01; and from 1.98 ± 0.79 to 2.63 ± 0.59; P < 0.01], while there were no significant changes in the plasma ANP and log-ANP levels during TH. In addition, the plasma BNP levels and log-BNP levels significantly decreased [from 450 (156, 1359) pg/mL to 127 (79, 429) pg/mL; P < 0.01; and from 2.63 ± 0.59 to 2.20 ± 0.57; P < 0.01] in the rewarming phase (Fig. 1a,b, Supplementary Figure S1). We added an analysis of the relationship between ANP and BNP, and thus found a positive relationship between these variables (r = 0.5542, P < 0.0001) (Supplementary Figure S2). Compared to the value at immediately before TH, the mean blood pressure (BP) was significantly decreased during TH and after rewarming [Pre-TH, 96 ± 21 mmHg; TH 12 hr, 82 ± 9.9 mmHg, P < 0.05 vs. Pre-TH; After rewarming, 78 ± 15 mmHg, P < 0.01 vs. Pre-TH] (Fig. 1c). The heart rate (HR) was significantly decreased during TH and significantly increased in the rewarming phase (Pre-TH, 94 ± 22 bpm; TH-12 hr, 76 ± 18 bpm, P < 0.01 vs. Pre-TH; After rewarming, 91 ± 13 bpm, P < 0.01 vs. TH 12 hr) (Fig. 1d). During TH, the diastolic pulmonary artery pressure (PAP) was significantly decreased, while there were no significant changes in the systolic PAP, cardiac index (CI), stroke volume index (SVI), or central venous pressure (CVP) (Fig. 1e,f) (Table 3). The CI and SVI were elevated in the rewarming phase, and the systemic vascular resistance index (SVRI) was elevated before TH (Pre-TH) and 12 h after the initiation of TH (TH 12 h), and subsequently decreased to the normal range in the rewarming phase (Table 3).

**Table 1:** Patients’ background characteristics.

Case	Gender	Age (years)	The cause of cardiac arrest	Comorbidities	In-hospital death
1	Male	51	Acute MI	CKD (HD), DM, pulmonary sarcoidosis	
2	Male	54	Acute MI	Schizophrenia	
3	Male	60	Acute MI	HT	
4	Male	57	CSA	HT, DM, sleep apnea syndrome	
5	Male	68	ALCAPA	None	
6	Male	87	Ischemic cardiomyopathy	Old MI	
7	Male	80	Acute MI	HT, DLP	〇
8	Male	51	Acute MI	Emphysema, DLP	
9	Male	46	Acute MI	Nephrotic syndrome, DM, HT, DLP	〇
10	Male	64	ACS	Old MI, CKD (HD), IgA nephropathy, HT	
11	Male	82	Unknown	Apical hypertrophic cardiomyopathy, gastric cancer (post surgery)	
12	Male	70	Acute MI	DM, HT	
13	Male	46	CSA	Paf, HT	
14	Female	51	Suspicion of cardiac sarcoidosis	HT, DLP	
15	Male	67	Unknown	Silent myocardial ischemia, HT	
16	Male	68	Acute MI	DM, DLP	
17	Male	45	Suspicion of DCM	None	
18	Female	48	Pulmonary thromboembolism	Deep-vein thrombosis, uterus adenomyosis	
19	Male	35	Suspicion of Brugada syndrome	Asthma	
20	Female	55	Unknown	CKD (HD), hypertensive disorders of pregnancy, Paf, post-parathyroidectomy, cancer of the uterine body (post surgery), DLP	
21	Male	71	Suspicion of CSA	esophageal stenosis (post balloon dilatation), hypothyroidism, DLP	

*MI* myocardial infarction, *CKD* chronic kidney disease, *HD* hemodialysis, *HT* hypertension, *DM* diabetes mellitus, *CSA* coronary spastic angina, *ALCAPA* anomalous left coronary artery from pulmonary artery, *DLP* dyslipidemia, *ACS* acute coronary syndrome, *DCM* dilated cardiomyopathy, *Paf* paroxysmal atrial fibrillation.

**Table 2:** Medicine and mechanical support administered during therapeutic hypothermia.

Case	NA (maximal dose, µg/min)	DOB	DOA	AMD	NTG	Nicardipine	Furosemide	Spironolactone (internal use)	Tolvaptan (internal use)	PCPS	IABP	CHD
1				〇	〇						〇	〇
2	〇 (6.7)											
3	〇 (6.7)	〇					〇					
4	〇 (3.3)											
5	〇 (5.0)			〇		〇						
6	〇 (8.3)	〇		〇							〇	
7	〇 (8.3)	〇	〇	〇						〇	〇	
8					〇	〇	〇					
9					〇	〇	〇	〇	〇			
10	〇 (5.0)	〇		〇	〇							〇
11	〇 (5.0)					〇						
12	〇 (3.3)					〇						
13	〇 (8.3)					〇		〇			〇	
14	〇 (3.3)			〇	〇			〇				
15						〇	〇					
16	〇 (5.0)				〇							
17	〇 (8.3)			〇	〇	〇						
18		〇			〇					〇	〇	
19	〇 (15)			〇								
20	〇 (3.3)			〇								〇
21	〇 (6.7)	〇			〇		〇			〇	〇	〇

*NA* noradrenaline, *DOB* dobutamine, *DOA* dopamine, *AMD* amiodarone, *NTG* nitroglycerin, *PCPS* percutaneous cardiopulmonary support device, *IABP* intra-aortic balloon pumping, *CHD* continuous hemodialysis.

**Table 3:** Comparison of the clinical data during therapeutic hypothermia.

	Pre-TH	TH 12 h	After rewarming
Deep body temperature (°C)	35.9 ± 1.5	34.0 ± 0.78**	36.4 ± 0.64^† †^
Mean BP, mmHg	96 ± 21	82 ± 9.9*	78 ± 15**
Heart rate, bpm	94 ± 22	76 ± 18**	91 ± 13^† †^
Systolic PAP, mmHg	33 ± 15	29 ± 12	30 ± 14
Diastolic PAP, mmHg	20 ± 8.5	16 ± 6.3*	16 ± 6.9*
CI, L/min/m^2^	2.4 ± 1.3	2.1 ± 0.78	2.9 ± 0.83^† †^
Stroke volume index, mL/m^2^	25 ± 12	29 ± 11	32 ± 11^† †^
CVP, mmHg	10.5 ± 5.3	10.1 ± 4.3	9.6 ± 5.1
SVRI, dyne-s/cm^5^/m^2^	3414 ± 1277	3218 ± 1116	2,122 ± 674**^,††^
ANP, pg/mL	44 (23, 158)	53 (31, 90)	56 (28, 94)
BNP, pg/mL	80 (21, 400)	450 (156, 1359)**	127 (79, 429)^† †^
Log ANP	1.75 ± 0.62	1.70 ± 0.38	1.72 ± 0.43
Log BNP	1.98 ± 0.79	2.63 ± 0.59**	2.20 ± 0.57*^,††^
Glucose, mg/dL	189 ± 65	197 ± 118	138 ± 54*^‚††^
Insulin, μU/mL	3.5 (1.2, 4.8)	9.0 (4.4, 16)**	7.6 (3.1, 14)**
Vasopressin, pg/mL	21 (16, 41)	9.2 (5.7, 25)	8.4 (4.4, 11)**
Cortisol, μg/dL	32 ± 7.1	28 ± 15	21 ± 9.8**
ACTH, pg/mL	85 (42, 176)	15 (4.5, 43)**	11 (2.4, 22)**

*TH* therapeutic hypothermia, *TH 12 h* 12 h after initiation of TH, *BP* blood pressure, *bpm* beats per minutes, *PAP* pulmonary artery pressure, *CI* cardiac index, *CVP* central venous pressure, *SVRI* systemic vascular resistance index, *ANP* A-type or atrial natriuretic peptide, *BNP* B-type or brain natriuretic peptide, *ACTH* adrenocorticotropic hormone.

*P < 0.05, **P < 0.01 vs. Pre-TH, ^†^P < 0.05, ††P < 0.01 vs. TH 12 h.

**Figure 2:**
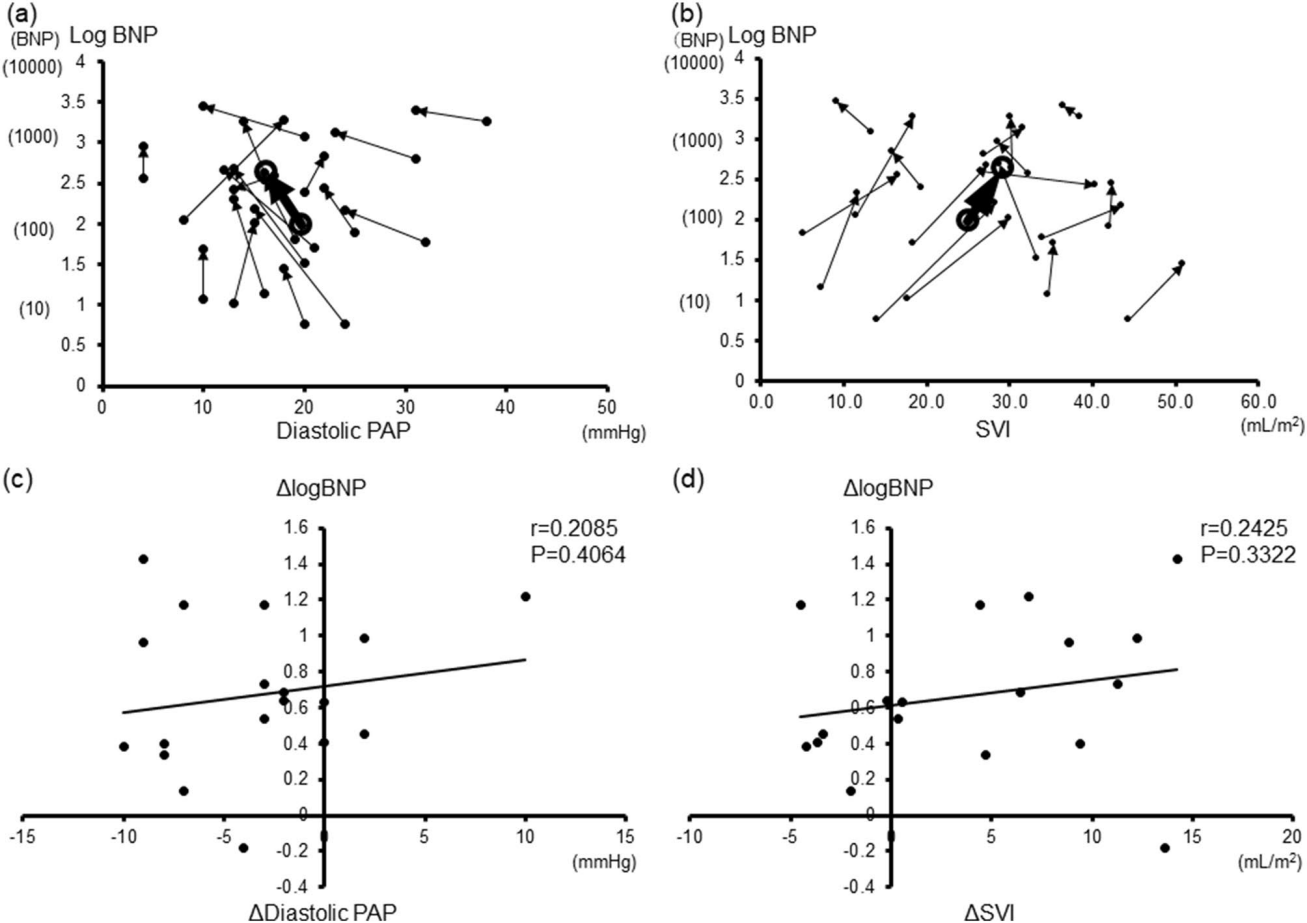
Variation in the relationship between the log-BNP levels and hemodynamic parameter during TH. The relationship between each patient's hemodynamics and the Log-BNP is indicated by an arrow as a change 12 h after TH. The average values are shown by the large symbols and heavy lines. **(a)** Diastolic PAP, **(b)** SVI. The relationship between the change in the log BNP levels versus the change in the hemodynamic variables. **(c)** Diastolic PAP. **(d)** SVI. *BNP* B-type or brain natriuretic peptide, *PAP* pulmonary artery pressure, *SVI* stroke volume index. *ΔDiastolic PAP* diastolic PAP at TH 12 h – diastolic PAP at pre-TH. *ΔSVI* SVI at TH 12 h – SVI at pre-TH.

Figure 2 shows the effects of TH on the relationship between the log-BNP and diastolic PAP (a) and SVI (b). At 12 h after the initiation of TH, the log-BNP levels were significantly increased, while the diastolic PAP was significantly decreased; there was no significant change in the SVI. Figure 2 shows the effects of TH on the relationship between the diastolic PAP and log-BNP and between the SVI and log-BNP. In both graphs, most plots moved significantly upward, either to the left or right (Fig. 2a,b). We also showed the relationship between the change in the log BNP levels (Δlog BNP) versus the change in hemodynamic variables (Δdiastolic PAP and Δ SVI) during TH. Regardless of the Δdiastolic PAP and Δ SVI, most log BNP values increased (i.e. Δlog BNP > 0) (Fig. 2c,d). These graphs indicated an increase in the plasma BNP levels during TH, regardless of the hemodynamics.

To clarify the relationship between the necessity of catecholamine (noradrenaline, dobutamine, and dopamine) and increasing BNP levels, we compared the %ΔBNP values between the patients who were treated with catecholamine (Catecholamine group) and the patients who were not treated with catecholamine (Non-catecholamine group). We found no difference between the two groups (Supplementary Table S1). The plasma BNP levels before TH (Pre-TH) did not differ between males and females, and the %ΔBNP of females tended to decrease in comparison to the values of males, although the difference was not statistically significant (P = 0.056) (Supplementary Table S2).

Supplementary Table S3 shows the results of the patients’ physical measurements and the catecholamine administered during TH. In this study, there was no relationship between BMI/BSA and the plasma BNP levels immediately before TH (Pre-TH), or between BMI/BSA and %ΔBNP, whereas the BMI/BSA values in the Non-catecholamine group were higher than those in the Catecholamine group (Supplementary Figure S3, Table S4).

## Discussion

Heart failure, renal failure, anemia, and inflammation have been suggested to be involved in BNP secretion, but there have been no data on the effects of hypothermia on BNP secretion. ANP has been shown to be secreted in cold environments in some human studies^17–19^. However, the ANP secretion may also depend on hemodynamics, and no report has clearly linked hypothermia and ANP secretion. The following points became clear in the present study: (1) The plasma BNP concentration is significantly increased in patients with cardiac arrest and return of spontaneous circulation (ROSC) during TH; (2) the BNP secretion is not related to hemodynamics, suggesting the possibility of stimulation by hypothermia; and (3) the BNP levels are significantly greater than those of ANP in cold environments.

An increase in plasma BNP levels is likely to lead to a warming effect. Previous reports demonstrated the presence of active brown adipose tissue (BAT) deposits in adult humans and suggested their role in controlling the body temperature in cold environments^22–25^. BAT plays an important role in regulating the energy balance and helps maintain the body temperature under hypothermic conditions in mammals. Activation of uncoupling protein 1 (UCP1), specifically expressed in BAT, increases glucose and free fatty acid (FFA) oxidation and enables mitochondrial uncoupled respiration, which leads to heat generation^13,26–28^. Bordicchia et al. reported that mice exposed to cold temperature had increased levels of circulating NPs as well as an increased expression of NP signaling receptor and a reduced expression of the NP clearance receptor in BAT and white adipose tissue (WAT). They also showed that, in human adipocytes, ANP and BNP activated the PPARγ coactivator-1α (PGC-1α) and UCP1 expression through a pathway from cGMP-dependent protein kinase (PKG) to p38 MAPK, and in mice, increased concentrations of NP promoted beige adipocyte development in WAT and increased thermogenic gene expression in BAT^28^. These data suggested that increases in the concentrations of NPs during cold exposure activates the browning system, perhaps in epicardial fat, to increase the energy expenditure without adversely affecting the heart or other tissues and thus protect the cardiac function. Further studies measuring cGMP levels and p38 MAPK activity in humans may show that this pathway-activation occurs during TH.

The present study showed that the plasma BNP levels significantly increased during TH without significantly affected the hemodynamics status, while the plasma ANP levels did not significantly change after TH. Previous reports have shown that cold exposure increased the plasma ANP levels in humans^17–19^. Although there was a positive correlation between the plasma ANP levels and the plasma BNP levels in this study, plasma ANP differs somewhat from plasma BNP with regard to its mechanisms of synthesis, secretion, and degradation^1,3,9,29,30^. For example, plasma ANP levels respond more quickly to pulmonary capillary wedge pressure (PCWP) than plasma BNP levels^30^. Differences in the background characteristics among cases may also influence plasma ANP levels. For example, although the subject of previous reports were healthy volunteers, the subjects in our study were patients who had achieved ROSC after cardiac arrest. Therefore, the elevation of the ANP levels during cold exposure in our study may have been masked by other factors, such as the fluctuating hemodynamic status. In addition, the impact of the cold environment on the atria and ventricles may differ. Our report thus suggests the potential to add “cold stimulation” as a new influential factor in basic research on BNP.

Hypothermia is one possible stimulus present during the complex after cardiopulmonary arrest resuscitation. We previously reported that the plasma level of BNP was significantly increased on admission in patients with acute myocardial infarction (AMI) and peaked at approximately 20 h after the onset^31^. In the present study, which included 8 patients with AMI, the sampling time of TH 12 h was 17 ± 1.7 h after the onset of AMI, which is different from the time required for BNP to reach its peak in our previous study^31^. In addition, in non-AMI patients in the present study, the BNP level increased and peaked at TH 12 h, supporting the notion that the elevation of the BNP level was due to hypothermia rather than the natural course of AMI in the present study.

Some reports have shown that BNP levels are increased under severe conditions, such as septic shock. Maeder et al. reported that increased BNP release due to neurohumoral activation other than myocyte stretching or impaired BNP clearance may occur in patients with septic shock^32^. Papanikolaou et al. also reported that the severity of critical illness, rather than septic cardiomyopathy, is probably the major determinant of BNP elevation in patients with critical sepsis, and they also showed in severe sepsis and septic shock patients that the BNP levels peaked on day 2 and decreased gradually thereafter^33^. Severe illness may thus influence the BNP levels in addition to the direct effect of TH. Nevertheless, we believe that the increased BNP levels are largely due to the effects of hypothermia, given the timing of initiating TH.

The sympathetic nervous system (SNS) and renin–angiotensin–aldosterone (RAA) systems are very important factors in the etiology of heart failure^34^. In addition to hemodynamic factors, neurohormonal factors—such as the SNS and RAA systems—stimulate NP secretion^35^. In this study, hemodynamic parameters that reflect the severity of heart failure, such as PA, SVI, and CI, were not observed to worsen during TH. We are certain that the plasma BNP levels were increasing during TH, but could not ascertain whether the increase in BNP during TH was due to TH itself or another factor accompanying TH (for example the SNS and RAS systems).

In the present study, the vasopressin, cortisol, and adrenocorticotropic hormone (ACTH) levels were significantly higher before TH than during or after TH. This is probably due to the effects of hemodynamic instability and stressful conditions. The detailed mechanism underlying the reduction in these hormone levels during TH and the rewarming process is currently unknown, and whether the decline is simply due to the course of nature or related to the effects of NP is a topic for further study.

In most patients of this study, the plasma BNP levels increased during TH. No apparent deterioration of heart failure was observed when we monitored PAP, SVI and CI, while the HR and BP decreased during TH. These reductions of HR and BP were probably due to hypothermia^36^. Furthermore, immediately after a return of spontaneous circulation (ROSC), most patients are hypotensive as a result of vasodilation from the post-resuscitation release of inflammatory factors and direct cardiac dysfunction from ischemia^36^; thus, they require catecholamine. In most of the patients in this study, catecholamines were started before therapeutic hypothermia. However, hypotension is one of the main side effects of Nesiritide, a recombinant BNP, with vasodilatory properties^37^, in the present study, we could not deny the possibility that hypotension and the need for catecholamine during TH were affected by plasma BNP, which is synthesized endogenously.

The target of this study were patients who survived after cardiac arrest, who were administered medicines that influence the BNP levels, such as steroids, carperitide, and catecholamine^38,39^. To improve accuracy, it is appropriate to exclude patients who were treated with these medicines. In the present study, the patients who received steroids and carperitide were a minority; thus, these patients were excluded. On the other hand, most patients were treated with catecholamine during TH; thus, it is difficult to exclude the patients who were treated with catecholamine. To clarify the relationship between the necessity of catecholamine and increasing BNP levels, we compared the %ΔBNP values between the Catecholamine group and the Non-catecholamine group; however, they did not differ to a statistically significant extent.

Although a previous study reported that females were more likely to have elevated BNP levels, probably due to the hormonal regulation of estrogen^40^, the plasma BNP levels before TH (Pre-TH) were did not differ between males and females in this study. The discrepancy between the previous study and the present study was probably due to the relatively small study population, the presence of a sex bias (3 females, 18 males), and because more of the patients in this study developed severe heart failure in comparison to the previous study. On the other hand—although the difference was not statistically significant—the %ΔBNP values of females tended to be lower than those of males. It is difficult to elucidate the precise mechanism of the sex differences in %ΔBNP during TH; however, we hypothesized that the BNP secretion during TH was stimulated by unconventional factors, such as cold exposure, which are not present during usual care.

Although, some previous studies reported that plasma BNP levels tend to be lower in obese patients^40,41^, we could not recognize a relationship between BMI/BSA and the plasma BNP levels immediately before TH (Pre-TH) in this study. There are some reasons for this discrepancy. The study population of the present study was very small, and the patients were evaluated at times when their condition was more severe in comparison to patients in other studies (e.g., immediately after ROSC). Similarly, there was no relationship between BMI/BSA and %ΔBNP, whereas the BMI/BSA values in the Non-catecholamine group were higher than those in the Catecholamine group. However, the precise reason for this difference is unknown.

### Study limitations

Several limitations associated with the present study warrant mention. This is a retrospective and small sample study conducted at a single center. We perform TH for all patients with cardiac arrest and ROSC unless there is a specific contraindication in our center. Therefore, we included no control group in this study. Given these points, this study is thus considered to be a useful preliminary study that shows how hypothermia affects an elevation of the BNP levels. As a next step, it will be necessary to collaborate with hospitals that do not use hypothermia, and to compare the changes in the plasma BNP levels after cardiac arrest without hypothermia. On the other hand, we are also planning to conduct animal experiments to investigate these aspects. In addition, we were unable to rule out the effect of certain unidentified confounders on the severe illness condition, such as cardiac arrest and ROSC, as discussed above.

## Conclusions

In conclusion, this study showed that the plasma BNP levels significantly increased more than five-fold during TH, with no relation to the hemodynamics. This elevation of BNP levels seems may have been influenced by hypothermia. Clarifying the underlying molecular mechanism may aid in the development of new therapeutic approaches.

## Methods

### Patient population

Between August 2018 and August 2019, we consecutively screened patients ≥ 18 years of age who were unconscious on admission to the hospital after out-of-hospital cardiac arrest of presumed cardiac cause, irrespective of the initial rhythm, and who had been treated with TH. The main exclusion criteria of TH were a tympanic membrane temperature below 30 °C, comatose status before cardiac arrest, pregnancy, terminal illness or patients for whom intensive care did not seem to be appropriate, and inherited blood coagulation disorders^21,36^.

In the trial period, we included all patients with cardiac arrest who underwent TH, except for those who received steroids and carperitide during TH. In the present study, however, most patients were treated with catecholamine, so we did not exclude these patients.

Clinical investigations were conducted in accordance with the principles expressed in the Declaration of Helsinki. The study was approved by the medical ethics committee of the Jikei University School of Medicine (31–223 9722). The Ethical Committee waived the need for informed written consent, since it was a retrospective study. Instead of obtaining informed consent from each patient, we posted a notice about the study design and contact information at a public location in our institution according to our routine ethical regulations. In this public notification, we ensured the opportunity for patients to refuse participation (opt-out) in this study.

### TH

There are four stages of TH: initiation, maintenance, rewarming, and return to normothermia. TH were initiated as soon as possible after ROSC with a target temperature of 34 °C. Our institution uses surface-cooling methods, including ice packs and cooling blankets, to maintain the target temperature from the rapid initiation of TH through the rewarming phase. Rewarming began 12 to 24 h after the initiation of cooling. Rewarming should be performed slowly, with a target rate of 0.25 ℃ every hour until the patient returns to normothermia (37 ℃), which can take 12 h^36^.

### Data collection

Data regarding the patients’ backgrounds and clinical findings were retrospectively collected from their medical records. We collected blood samples and hemodynamic data, including the PAP and CI, with a Swan-Ganz catheter at the following 3 points: immediately before TH (Pre-TH), 12 h after initiation of TH (TH 12 h), and the end of rewarming (After rewarming). In principle, a Swan-Gantz catheter was routinely inserted into the femoral or internal jugular vein after admission and was kept in place during TH in all patients. The CI was measured by the thermodilution technique. Biochemical analyses of plasma and serum were performed to determine the levels of Hb, eGFR, ANP, BNP, glucose, insulin, cortisol, ACTH, and vasopressin. The LVEF were measured by TTE at the time of CCU admission. ΔDiastolic PAP (or ΔSVI) was defined as the diastolic PAP (or ΔSVI) at TH 12 h minus the baseline level (at Pre-TH) for each patient. At 12 h after the initiation of TH (TH 12 h), we defined the percentage change from baseline in BNP as %ΔBNP [%ΔBNP (%) = 100 × (the plasma BNP level at TH 12 h − the plasma BNP level at Pre-TH)/the plasma BNP level at Pre-TH].

### Natriuretic peptide measurements

To measure the plasma ANP levels, blood samples were collected in tubes containing aprotinin (500 KIU/mL) and EDTA (ethylenediaminetetraacetic acid)-2Na (1.5 mg/mL), and then separately and without delay they were centrifuged at 3000 rpm for 10 min at 4 °C, and then frozen and stored at − 20 °C until ANP measurements were performed. The ANP plasma levels were measured using a Determiner CL ANP (Hitachi Chemical Diagnostics Systems Co., Ltd., Tokyo, Japan), with the measurement outsourced to a commercial clinical laboratory. According to the package insert of the ANP assay, ANP is measured by a chemiluminescent enzyme immunoassay method using two different types of anti-Human Atrial Peptide antibody, and the intra-assay coefficient of variation (CV) was ≤ 15% (10–60 pg/mL) and ≤ 10% (61–1000 pg/mL), respectively. To measure the plasma BNP levels, blood samples were collected in tubes containing EDTA, and then were immediately centrifuged at 3000 rpm for 5 min at 19 °C. Thereafter, the BNP plasma levels were immediately measured by an Architect BNP-JP assay (Abbott Laboratories, Abbott Park, IL, USA), which had been used in the clinical laboratory of our institution as a routine assay. In the BNP assay, the sample and anti-BNP-coated paramagnetic microparticles were incubated in the first step, followed by sandwich formation with anti-BNP acridinium-labeled conjugate in the second step. The chemiluminescent reaction with acridinium, which shows a direct relationship with the amount of BNP in the sample, was conducted. According to the manufacturer, the quality of the Architect BNP assay was continually assessed internally using control samples consisting of three levels (low, medium and high) prior to shipping in order to ensure it did not deviate from the pre-defined performance shown in the package insert^42,43^.

### Statistical analyses

Data were expressed as the mean ± standard deviation (SD) or the median (25th, 75th percentile) for significantly skewed variables. The blood sample data and hemodynamic data were compared among three time points using a repeated measure analysis of variance followed by Bonferroni’s test. However, when the variable was significantly skewed, the Friedman test was performed. The Wilcoxon signed rank test was used when the same patients were evaluated at two different points. The differences in continuous variables between two groups were evaluated by the unpaired Student t-test or Man-Whitney rank-sum test. The correlation between the ANP and BNP levels was investigated by a simple regression analysis and expressed as Spearman’s correlation coefficient. When we assessed the correlation between the change in BNP and the change in hemodynamic variables (such as diastolic PAP and SVI), BNP was logarithmically transformed for inclusion in linear models because of its exponential change. All statistical analyses were performed using the STATA statistic software program (version 14.0, STATA Corp., College Station, TX, USA) or the SPSS statistics software program (version 24.0, SPSS Inc., Chicago, IL, USA). P values of < 0.05 were considered to indicate statistical significance.

## Supplementary information

Supplementary Information.
