# Supplementary material for: Therapeutic hypothermia after cardiac arrest increases the plasma level of B-type natriuretic peptide
Source: Sci Rep. 2020 Sep 23;10:15545. doi: 10.1038/s41598-020-72703-2 (PMC7511910; doi:10.1038/s41598-020-72703-2)
Supplement: Supplementary file 1 — Supplementary Information. [file 41598_2020_72703_MOESM1_ESM.docx]

**Supplementary Information**

**Therapeutic Hypothermia after Cardiac Arrest Increases the Plasma Level of B-type Natriuretic Peptide**

Yusuke Kashiwagi* ^1,2^, Kimiaki Komukai ^1^, Haruka Kimura ^2^, Toraaki Okuyama ^1^, Tomoki Maehara ^1^, Keisuke Fukushima ^1^, Takahito Kamba ^1^, Yoshitsugu Oki ^1^, Keisuke Shirasaki ^1^, Takeyuki Kubota ^1^, Satoru Miyanaga ^1^, Tomohisa Nagoshi ^2^, Michihiro Yoshimura ^2^

^1^ Division of Cardiology, Department of Internal Medicine, The Jikei University School of Medicine, Kashiwa Hospital, Kashiwa, Chiba, Japan

^2^ Division of Cardiology, Department of Internal Medicine, The Jikei University School of Medicine, Tokyo, Japan

Supplementary Table S1: The %ΔBNP values of the Non-catecholamine group and the Catecholamine group.

|  | Non-catecholamine group  (n=4) | Catecholamine group  (n=17) | P |
| --- | --- | --- | --- |
| %ΔBNP (%) | 331 (181, 440) | 243 (151, 873) | 0.7202 |

BNP, B-type or brain natriuretic peptide; TH, therapeutic hypothermia

%ΔBNP (%) =100 × (the plasma BNP level at TH 12hr - the plasma BNP level at Pre-TH) / the plasma BNP level at Pre-TH.

Supplementary Table S2: The %ΔBNP values of females and males.

|  | Female (n=3) | Male (n=18) | P |
| --- | --- | --- | --- |
| The plasma BNP (Pre-TH) (pg/mL) | 362 (234, 630) | 62 (14, 400) | 0.191 |
| %ΔBNP (%) | 116 (92, 155) | 362 (184, 873) | 0.056 |

BNP, B-type or brain natriuretic peptide; TH, therapeutic hypothermia

%ΔBNP (%) =100 × (the plasma BNP level at TH 12hr - the plasma BNP level at Pre-TH) / the plasma BNP level at Pre-TH.

Supplementary Table S3: The physical measurements of patients and the administration of catecholamine during therapeutic hypothermia.

| **Case** | **Age　(years)** | **Height (m)** | **BW (kg)** | **BMI (kg/m^2^)** | **BSA(m^2^)** | **Noradrenaline** | **DOB** | **DOA** |
| --- | --- | --- | --- | --- | --- | --- | --- | --- |
| 1 | 51 | 1.70 | 65.4 | 22.63 | 1.76 |  |  |  |
| 2 | 54 | 1.69 | 60.0 | 21.01 | 1.69 | 〇 |  |  |
| 3 | 60 | 1.60 | 60.0 | 23.44 | 1.62 | 〇 | 〇 |  |
| 4 | 57 | 1.78 | 90.0 | 28.41 | 2.08 | 〇 |  |  |
| 5 | 68 | 1.76 | 63.0 | 20.34 | 1.77 | 〇 |  |  |
| 6 | 87 | 1.62 | 50.4 | 19.20 | 1.52 | 〇 | 〇 |  |
| 7 | 80 | 1.78 | 67.0 | 21.15 | 1.84 | 〇 | 〇 | 〇 |
| 8 | 51 | 1.70 | 70.0 | 24.22 | 1.81 |  |  |  |
| 9 | 46 | 1.70 | 110.0 | 38.06 | 2.19 |  |  |  |
| 10 | 64 | N/A | N/A | N/A | N/A | 〇 | 〇 |  |
| 11 | 82 | 1.60 | 55.0 | 21.48 | 1.56 | 〇 |  |  |
| 12 | 70 | 1.64 | 56.0 | 20.82 | 1.60 | 〇 |  |  |
| 13 | 46 | 1.72 | 66.0 | 22.31 | 1.78 | 〇 |  |  |
| 14 | 51 | 1.56 | 59.0 | 24.24 | 1.58 | 〇 |  |  |
| 15 | 67 | 1.61 | 94.0 | 36.26 | 1.97 |  |  |  |
| 16 | 68 | 1.68 | 58.0 | 20.55 | 1.66 | 〇 |  |  |
| 17 | 45 | 1.65 | 72.0 | 26.45 | 1.79 | 〇 |  |  |
| 18 | 48 | 1.54 | 45.0 | 18.97 | 1.40 |  | 〇 |  |
| 19 | 35 | 1.74 | 70.0 | 23.12 | 1.84 | 〇 |  |  |
| 20 | 55 | 1.57 | 59.1 | 23.98 | 1.59 | 〇 |  |  |
| 21 | 71 | 1.70 | 63.0 | 21.80 | 1.73 | 〇 | 〇 |  |

BW, body weight; BMI; body mass index; BSA, body surface area; N/A, not available; DOB, dobutamine; DOA, dopamine.

Supplementary Table S4: The BMI/BSA values of the patients in the Non-catecholamine group and the Catecholamine group.

|  | Non-catecholamine group  (n=4) | Catecholamine group  (n=17) | P |
| --- | --- | --- | --- |
| BMI (kg/m^2^) | 30.2 (23.4, 37.2) | 21.6 (20.7, 23.7) | 0.030 |
| BSA (m^2^) | 1.93 ± 0.20 | 1.69 ± 0.16 | 0.019 |

BW, body weight; BMI; body mass index; BSA, body surface area.

Supplementary Figure Legends

**Supplementary Figure S1. The percentage change form baseline in NP levels during TH.**

Plotting the percentage changes from baseline in the NP levels during TH. %ΔBNP was defined as follows: [%ΔBNP (%) =100 × (the plasma BNP level at TH 12hr - the plasma BNP level at Pre-TH) / the plasma BNP level at Pre-TH].

NP, natriuretic peptide; TH, therapeutic hypothermia.

**Supplementary Figure S2: The associations between the ANP and BNP levels.**

Results of simple regression analyses between the ANP and BNP levels during TH are shown.

ANP, A-type or atrial natriuretic peptide; BNP, B-type or brain natriuretic peptide; TH, therapeutic hypothermia.

**Supplementary Figure S3. The association between BMI/BSA and BNP (Pre-TH), and between BMI/BSA and %ΔBNP (%).**

The results of regression analyses between BMI and BNP (Pre-TH) (a), between BSA and BNP (Pre-TH) (b), between BMI and %ΔBNP (c) and between BSA and %ΔBNP (d) are shown.

BW, body weight; BMI; body mass index; BSA, body surface area; BNP, B-type or brain natriuretic peptide; Pre-TH, immediately before therapeutic hypothermia.

**Supplementary Figure S1**


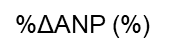


Pre-TH

TH 12hr

After rewarming


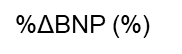


TH 12hr

After rewarming

Pre-TH

After rewarming

**Supplementary Figure S2**


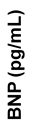


*r*＝0.554

*P*<0.0001

**ANP (pg/mL)**

pg/mL

**Supplementary Figure S3**

**
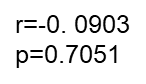

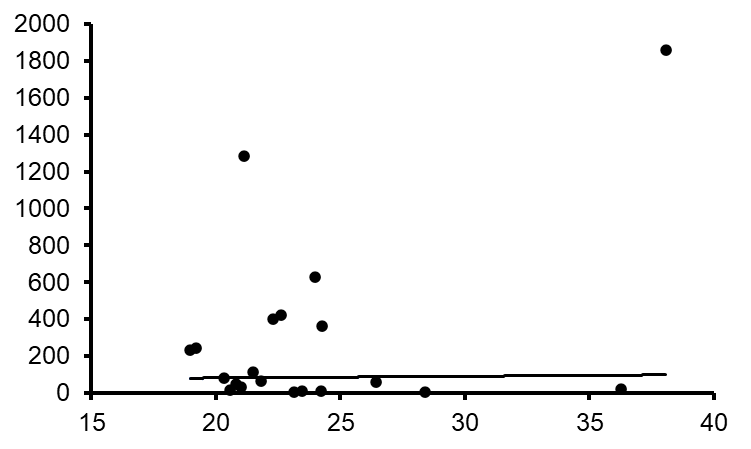
**

(a)

**
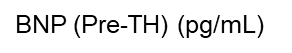
**

(kg/m^2^)

**BMI**

(b)

**
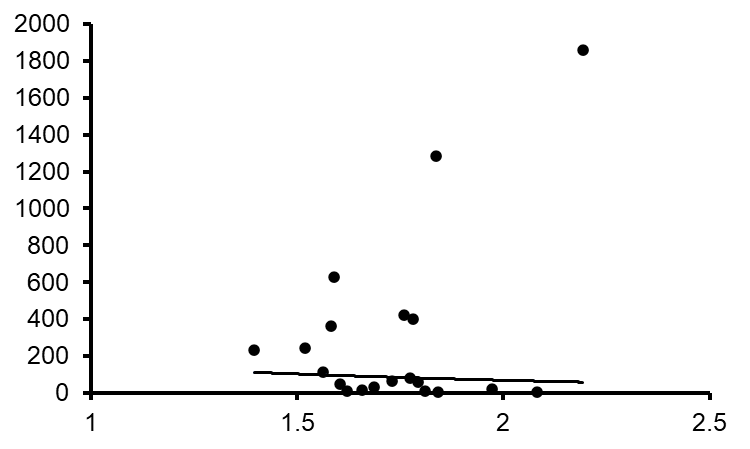
**

**
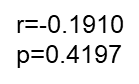
**

**
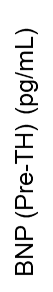
**

(m^2^)

**BSA**

(c)

r=-0.1459

p=0.5395

**
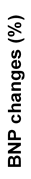
**

%ΔBNP (%)

(kg/m^2^)

**BMI**

(d)

**
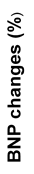
**

%ΔBNP (%)

(m^2^)

**BSA**

r=-0.0105

p=0.9649

(m^2)^

BSA

r=-0.0105

p=0.9649
